# Genome-Wide Identification, Molecular Evolution, and Expression Profiling Analysis of Pectin Methylesterase Inhibitor Genes in *Brassica campestris* ssp. *chinensis*

**DOI:** 10.3390/ijms19051338

**Published:** 2018-05-02

**Authors:** Tingting Liu, Hui Yu, Xingpeng Xiong, Xiaoyan Yue, Youjian Yu, Li Huang, Jiashu Cao

**Affiliations:** 1 Laboratory of Cell and Molecular Biology, Institute of Vegetable Science, Zhejiang University, Hangzhou 310058, China; 11416009@zju.edu.cn (T.L.); 21616082@zju.edu.cn (H.Y.); 11216053@zju.edu.cn (X.X.); 11516008@zju.edu.cn (X.Y.); lihuang@zju.edu.cn (L.H.); 2 Key Laboratory of Horticultural Plant Growth, Development and Quality Improvement, Ministry of Agriculture, Hangzhou 310058, China; 3 Zhejiang Provincial Key Laboratory of Horticultural Plant Integrative Biology, Hangzhou 310058, China; 4 Department of Horticulture, College of Agriculture and Food Science, Zhejiang A & F University, Lin’an 311300, China; yjyu@zafu.edu.cn

**Keywords:** *Brassica campestris*, PMEIs, evolution, expression patterns

## Abstract

Pectin methylesterase inhibitor genes (*PMEIs*) are a large multigene family and play crucial roles in cell wall modifications in plant growth and development. Here, a comprehensive analysis of the PMEI gene family in *Brassica* *campestris*, an important leaf vegetable, was performed. We identified 100 *Brassica* *campestris* *PMEI* genes (*BcPMEIs*), among which 96 *BcPMEIs* were unevenly distributed on 10 chromosomes and nine tandem arrays containing 20 *BcPMEIs* were found. We also detected 80 pairs of syntenic PMEI orthologs. These findings indicated that whole-genome triplication (WGT) and tandem duplication (TD) were the main mechanisms accounting for the current number of *BcPMEIs*. In evolution, *BcPMEIs* were retained preferentially and biasedly, consistent with the gene balance hypothesis and two-step theory, respectively. The molecular evolution analysis of *BcPMEIs* manifested that they evolved through purifying selection and the divergence time is in accordance with the WGT data of *B. campestris*. To obtain the functional information of *BcPMEIs*, the expression patterns in five tissues and the *cis*-elements distributed in promoter regions were investigated. This work can provide a better understanding of the molecular evolution and biological function of *PMEIs* in *B. campestris*.

## 1. Introduction

The plant cell wall is a highly complex structure mainly containing polysaccharides and proteins and is indispensable to the proper plant growth and development [1]. The polysaccharides are mostly made up of cellulose, hemicellulose, and pectin, with pectin the major component [2]. Pectin arose after the separation between chlorophyta and charophyce and is essential for cellular structural integrity, cell adhesion, and the mediation of defense responses [3,4,5,6]. It can be divided into homogalacturonan (HG), xylogalacturonan (XGA), rhamnogalacturonan I (RGI), and rhamnogalacturonan II (RGII) based on the different structures [7]. The four different pectin domains are linked to each other, constituting a huge network [3]. HG, the most abundant pectic polysaccharide, is methyl-esterified in medial-Golgi; then the methyl-esterified HG is conveyed to the cell wall and proceeds with demethylation by pectin methylesterases (PMEs) [3]. PMEs belong to a big hydrolase family and are regulated by pectin methylesterase inhibitors (PMEIs) [2]. The proteins of PME and PMEI can form a reversible 1:1 complex, but the PMEIs only inhibit plant PMEs and do not affect the PME activity of microbe origin [8].

PMEIs play important roles in many stages of plant growth, development, and defense, such as pollen maturation and pollen tube growth, seed germination, hypocotyl elongation, fruit ripening, and plant immunity [9,10,11,12,13]. *Bra014099*, *Bra019903*, and *Bra032239* might be involved in male sterile [14]. *AtPMEI3* has been proposed to regulate the primordia formation in *Arabidopsis* inflorescence meristems [15]. *AtPMEI6*, specially expressed in the seed coat, can affect the speed of seed mucilage [16]. *SolyPMEI*, a tomato PMEI gene, can influence fruit softening and ripening by regulating the methylesterification of pectin [11]. *OsPMEI28* is demonstrated to be a critical structural modulator and overexpression of *OsPMEI28* in rice leads to dwarf phenotypes and reduced culm diameter [17]. *CaPMEI1*, a PMEI gene in pepper, is involved in fungal resistance, as well as drought and oxidative stress tolerance [18]. During *Botrytis* attack, AtPMEI10, AtPMEI11, and AtPMEI12 can be induced to fight against the infection [13]. 

Gene duplication is recognized as a common phenomenon in the evolution of plants and can provide raw materials for adaptive evolution [19]. Several modes of gene duplication, such as WGD and tandem duplication (TD), have been summarized by a previous study, among which WGD, a prevalent and recurring event, plays a significant role in the evolution of higher eukaryotes [20,21]. The Brassicaceae are a good model for studying the polyploidy and evolution [22]. Approximately 338 genera and 3709 species worldwide with major scientific and economic importance belong to Brassicaceae, including some model species, such as *Arabidopsis* and *Brassica* [23,24]. *Arabidopsis*, the well-known model plant, has experienced three whole-genome duplication (WGD) events [25]. *Brassica campestris* (synonym of *Brassica rapa*), an economically important vegetable crop, is a diploid *Brassica* species [26]. Besides the three WGD events shared with *Arabidopsis*, *B. campestris* experienced another whole-genome triplication (WGT) event that led to its separation from *Arabidopsis* 13–17 million years ago (MYA) [27]. The divergence time, which is short enough for most *B. campestris* genes to be identified in *Arabidopsis* but long enough for the genome to be fractionated, makes *B. campestris* ideal for the study of gene families [26,28]. 

The genome-wide analyses of PMEIs were performed in many plants, such as *Arabidopsis*, rice, and flax, with 78, 49, and 95 members identified, respectively [29,30,31], indicating that PMEIs belong to a large multigene family, like PMEs. In this study, 100 *B. campestris PMEI* genes (*BcPMEIs*) were identified and characterized. Comprehensive studies of the PMEI gene family in *B. campestris* were conducted, including phylogenetic tree, gene structures, physicochemical properties, chromosomal locations, conserved motifs, and molecular evolution. Additionally, we carried out the analysis of synteny and retention rate between *BcPMEIs* and *Arabidopsis thaliana PMEI* genes (*AtPMEIs*) to reveal the impacts of WGT on the evolution of *BcPMEIs*. To obtain the functional information of *BcPMEIs*, their expression patterns in five tissues were analyzed by quantitative real-time (qRT) PCR. The *cis*-elements detected in promoter regions suggested that *BcPMEIs* might be involved in hormone regulation and stress tolerance. Our findings can provide valuable insights for studying the molecular evolution and biological functions of *PMEIs* in *B. campestris*.

## 2. Results

### 2.1. Identification and Phylogenetic Analysis of PMEI Gene Family in B. campestris

A total of 165 PMEI gene members were found by the HMMER 3.0 software with default values. In addition, 196 PMEIs were acquired from the BLASTP searches in BRAD by using 78 AtPMEIs and 49 *Oryza sativa* PMEIs (OsPMEIs) as queries. After removing the redundant sequences obtained from the above two methods, SMART and Pfam were adopted to further verify the candidates whether or not cover the conserved PMEI domain (PF04043). Finally, 100 PMEI members were identified in the whole genome of *B. campestris* (Table S1).

In the phylogenetic tree, the 100 *BcPMEIs* were classified into five clades, containing 19, 6, 16, 25, and 34 members, respectively (Figure S1). However, in accordance with the research results of PMEI gene family in flax [30] and rice [29], we failed to find any common sequence features that can distinguish the subclassifications of *BcPMEIs* from each other.

### 2.2. Characterization of Gene Structure, Conserved Motif, and Physicochemical Properties

**Figure 1:**
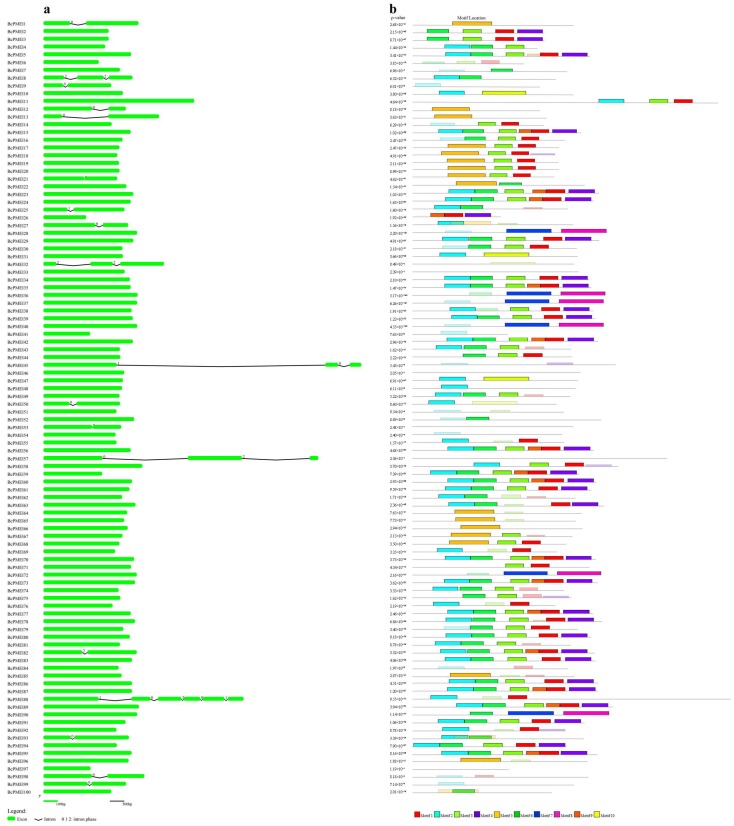
Gene structures and conserved motifs of *PMEIs* in *Brassica campestris*. (**a**) Exon–intron organization of *BcPMEIs*. The black lines and green boxes stand for introns and exons, respectively; (**b**) Distributions of conserved motifs in BcPMEIs. The differently colored boxes, numbered 1–10 at the bottom, denote differently conserved motifs.

The exon–intron structural analysis of *BcPMEIs* was performed by comparing the coding sequences with their corresponding genomic DNA sequences. The results showed that most of the *BcPMEIs* (88/100) only contain exon in their DNA sequences (Figure 1a), which was in line with previous studies [29,32]. Among the remaining 12 *BcPMEIs*, their coding sequences were disrupted by one to five introns, and a total of 26 introns were detected with 54%, 15%, and 31% in phases 0, 1 and 2, respectively. In order to detect the motifs shared among the PMEIs in *B. campestris*, we identified 10 conserved motifs using the MEME program (Figure 1b). The amino acid number of the motifs ranged from 15 (motif 9) to 50 (motifs 7, 8, and 10). Each BcPMEI protein covered 0 to 6 conserved motifs and the number of site distribution of motifs ranged from three (motif 10) to 50 (motifs 1, 2, and 3) (Figure S2). The length of the coding sequences of *BcPMEIs* ranged from 297 bp (*BcPMEI26*) to 1053 bp (*BcPMEI88*), and the number of the amino acids they encoded was from 98 to 350 with the predicted molecular weight from 10.9 to 38.2 kDa (Table S1). The theoretical isoelectric point varied from 4.06 (*BcPMEI11*) to 10.61 (*BcPMEI41*). A total of 82 BcPMEIs covered a signal peptide sequence with 16 to 31 amino acids in length, and 28 BcPMEIs had a transmembrane helix. Plant-mPLoc predicted that 97 BcPMEIs were secreted to the cell membrane. Based on the predictions of signal peptide, transmembrane domain, and subcellular localization, 98 BcPMEIs are predicted to be extracellular and two BcPMEIs were predicted to be localized to the nucleus, consistent with the subcellular localization results of PMEIs in flax [30].

### 2.3. Chromosomal Distribution of PMEIs in B. campestris

**Figure 2:**
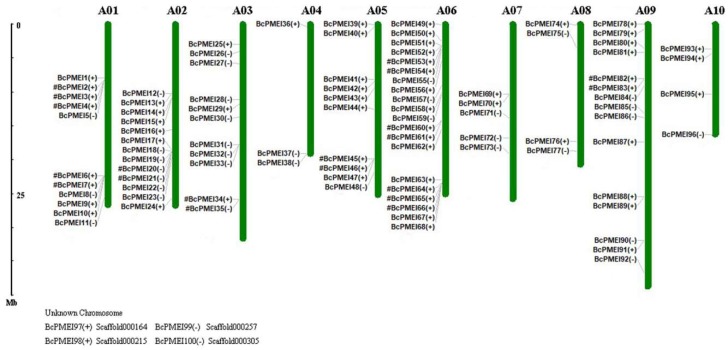
Chromosomal locations of *PMEIs* in *Brassica campestris.* The chromosome numbers are represented on the top of each chromosome. The positive (+) and negative (−) signs indicate forward and reverse orientation, respectively. The names of tandem genes lie next to pound signs (#). Four *BcPMEIs* could not be mapped onto a specific chromosome.

A total of 96 *BcPMEIs,* namely *BcPMEI1*-*BcPMEI96*, were located on 10 chromosomes with an obviously uneven distribution and the other four unmapped genes, namely *BcPMEI97*–*BcPMEI100*, were distributed on scaffold000164, scaffold000215, scaffold000257, and scaffold000305, respectively (Figure 2). The maximum number of *BcPMEIs* was discovered on chromosome 6 with up to 20 while the minimum number was on chromosome 4 with only three. Chromosomes 1, 2, 3, and 9 included more than 10 *BcPMEIs* with 11, 13, 11, and 15 genes located, respectively, while chromosomes 5, 7, 8, and 10 only contained 10, five, four, and four genes, respectively. Sixty-one *BcPMEIs* were present in the 5’ to 3’ direction, and the remaining members were at the opposite direction. In addition, among the *BcPMEIs* located on the 10 *B. campestris* chromosomes, nine tandemly duplicated clusters were identified, which were mapped on chromosome 1 (*BcPMEI2*/*BcPMEI3*/*BcPMEI4*; *BcPMEI6*/*BcPMEI7*), chromosome 2 (*BcPMEI20*/*BcPMEI21*), chromosome 3 (*BcPMEI34*/*BcPMEI35*), chromosome 5 (*BcPMEI45*/*BcPMEI46*), chromosome 6 (*BcPMEI53*/*BcPMEI54; BcPMEI60*/*BcPMEI61; BcPMEI64*/*BcPMEI65*/*BcPME66*), and chromosome 9 (*BcPMEI82*/*BcPMEI83*), respectively.

### 2.4. Synteny and Retained Proportion Analysis

**Figure 3:**
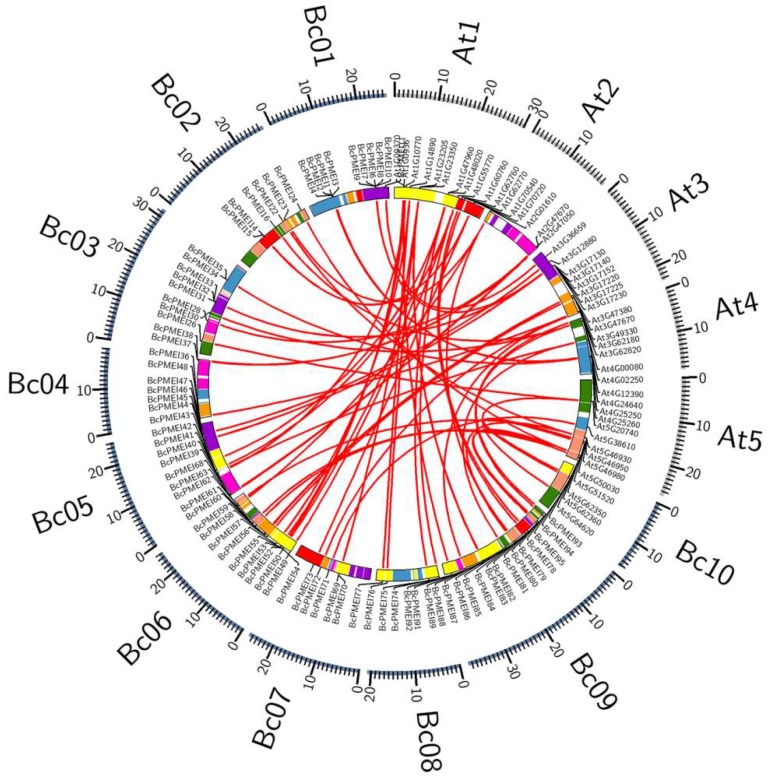
Syntenic analysis of *PMEIs* in *Brassica campestris* and *Arabidopsis thaliana*. The 24 genomic blocks are colored on the basis of the inferred ancestral chromosomes following an established convention.

On the basis of the well-conserved syntenic relationships between *B. campestris* and *A. thaliana*, we investigated the syntenic orthologous gene pairs between *BcPMEIs* and *AtPMEIs* by searching the “syntenic gene” in BRAD [33]. As shown in Figure 3, up to 80 pairs of orthologous *PMEIs* were detected in the same genomic blocks, implying that the expansion of *BcPMEIs* was mainly attributed to the WGT event. Additionally, by searching “nonsyntenic orthologous” in BRAD, we identified four pairs of nonsyntenic orthologous *PMEIs* (Table S2).

**Figure 4:**
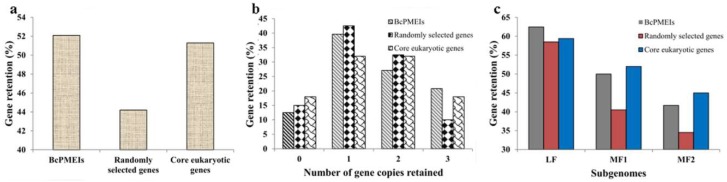
The retained rate of *PMEIs* in *Brassica campestris*. (**a**) Retained rates of *BcPMEIs*, randomly selected genes, and core eukaryotic genes; (**b**) retention rates by the number of homologous copies in the syntenic region; (**c**) retained rates of *BcPMEIs*, randomly selected genes, and core eukaryotic genes among the three subgenomes of *B. campestris*.

Seventeen loci of *AtPMEIs* did not have the orthologous gene in *B. campestris*. To know whether these loci were lost in *B. campestris* or were newly acquired in *Arabidopsis*, the analysis of their orthologous genes in other Brassicaceae species was performed on the basis of a previous study [34]. The results showed that 6 loci of *AtPMEIs* might be lost in *B. campestris* and 11 loci might arise after the divergence between *Arabidopsis* and *B. campestris* (Table S3)*.* Similarly, the 13 loci of *BcPMEIs* that have no orthologous gene in *Arabidopsis* were identified to appear after WGT. On the basis of these results, we computed the retention rate of *PMEIs* in *B. campestris* (Figure 4). Meanwhile, the retention of *BcPMEIs* was compared to that of a set of 458 core eukaryotic genes and 458 randomly selected genes [35]. The analysis results showed that 52% of *BcPMEIs* were reserved, similar to the retention of core eukaryotic genes (51%), but much higher than that of random genes (44%) (Figure 4a). Moreover, compared with the other two sets, fewer *BcPMEIs* (12.5%) were completely lost and more *BcPMEIs* (21%) retained three copies (Figure 4b).

To know the retained proportions of *BcPMEIs* in the whole genome level, we conducted the calculation in the three subgenomes, namely least fractionated (LF), medium fractionated (MF1), and most fractionated (MF2) subgenomes (Figure 4c) [26,36]. The PMEI gene family retained more members in the LF subgenome (60%) than those in MF1 and MF2 subgenomes (50% and 42%, respectively). In the LF subgenome, *BcPMEIs,* randomly selected genes, and core eukaryotic genes have similar retained rates. In the MF1 and MF2 subgenomes, *BcPMEIs* showed similar retained rates in comparison with the core eukaryotic genes, and both gene sets were retained in remarkably higher rates than the random gene set. So, the *BcPMEIs* was biasedly retained in the three subgenomes of *B. campestris*, and the highly reserved rate of *PMEIs* might mainly result from the gene retention in the LF subgenome.

### 2.5. Evolutionary Analysis of BcPMEIs

**Figure 5:**
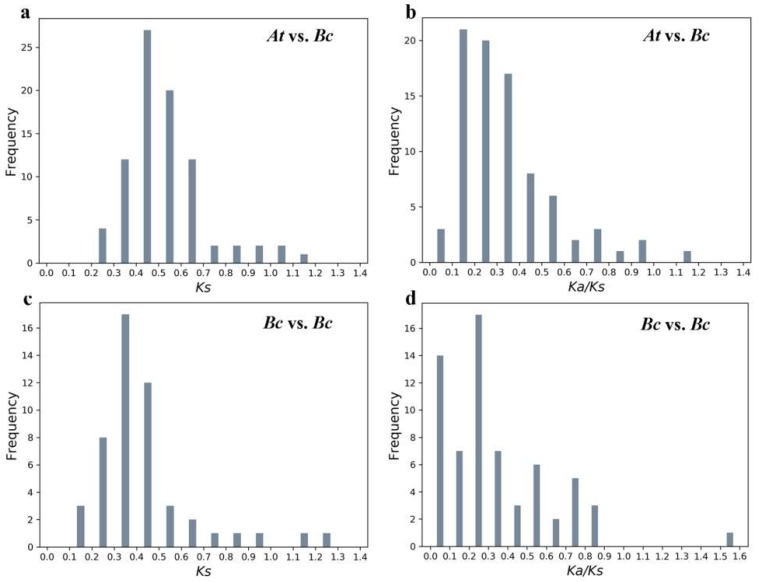
Frequency distributions of the *Ks* and *Ka*/*Ks* values of PMEI homolog pairs. (**a**,**b**) *Ks* and *Ka*/*Ks* value distributions of the PMEI ortholog pairs between the *Brassica campestris* and *Arabidopsis* genomes; (**c**,**d**) *Ks* and *Ka*/*Ks* value distributions of the PMEI paralog pairs in the *B. campestris* genome. The vertical axes represent the frequency of paired sequences and the horizontal axes represent the *Ks* and *Ka*/*Ks* values at a 0.1 interval.

The nonsynonymous substitution rate (*Ka*), synonymous substitution rate (*Ks*)*,* and ω ratio (*Ka*/*Ks*) of homogenous were computed to explore their selection types. The *Ks*, as the proxy for time, was usually used to measure the divergence time. A total of 84 PMEI orthologous gene pairs were analyzed (Table S4). Their *Ks* values were from 0.30 to 0.70 and were concentrated on 0.50 (Figure 5a), with a duplication time of about 16.67 MYA, close to the WGT time of *B. campestris* (13–17 MYA) [26]. The ω ratios of 83 ortholog pairs were lower than 1 (Figure 5b), implying that they experienced purify selection except for *BcPMEI98*. Furthermore, only three pairs might be under strong purifying selection stress with a ω ratio lower than 0.1, leading to the relatively similar functions of these orthologous gene pairs [22]. Among the three subgenomes, the *Ks* values and ω ratios of orthologous gene pairs did not have a significant difference (Figure S3). In addition, the *Ks* values of 52 BcPMEI paralog pairs ranged from 0.13 to 1.99, with an average *Ks* value of 0.49 (Figure 5c, Table S5). So, the average duplication time of paralog pairs was 16.17 MYA, in accordance with the *B. campestris* WGT date. The ω ratios of most (51/52) paralog pairs were lower than 1, suggesting that they evolved through purifying selection (Figure 5d).

### 2.6. Expression Analysis of BcPMEIs in Different Tissues and Promoter::GUS Fusions

**Figure 6:**
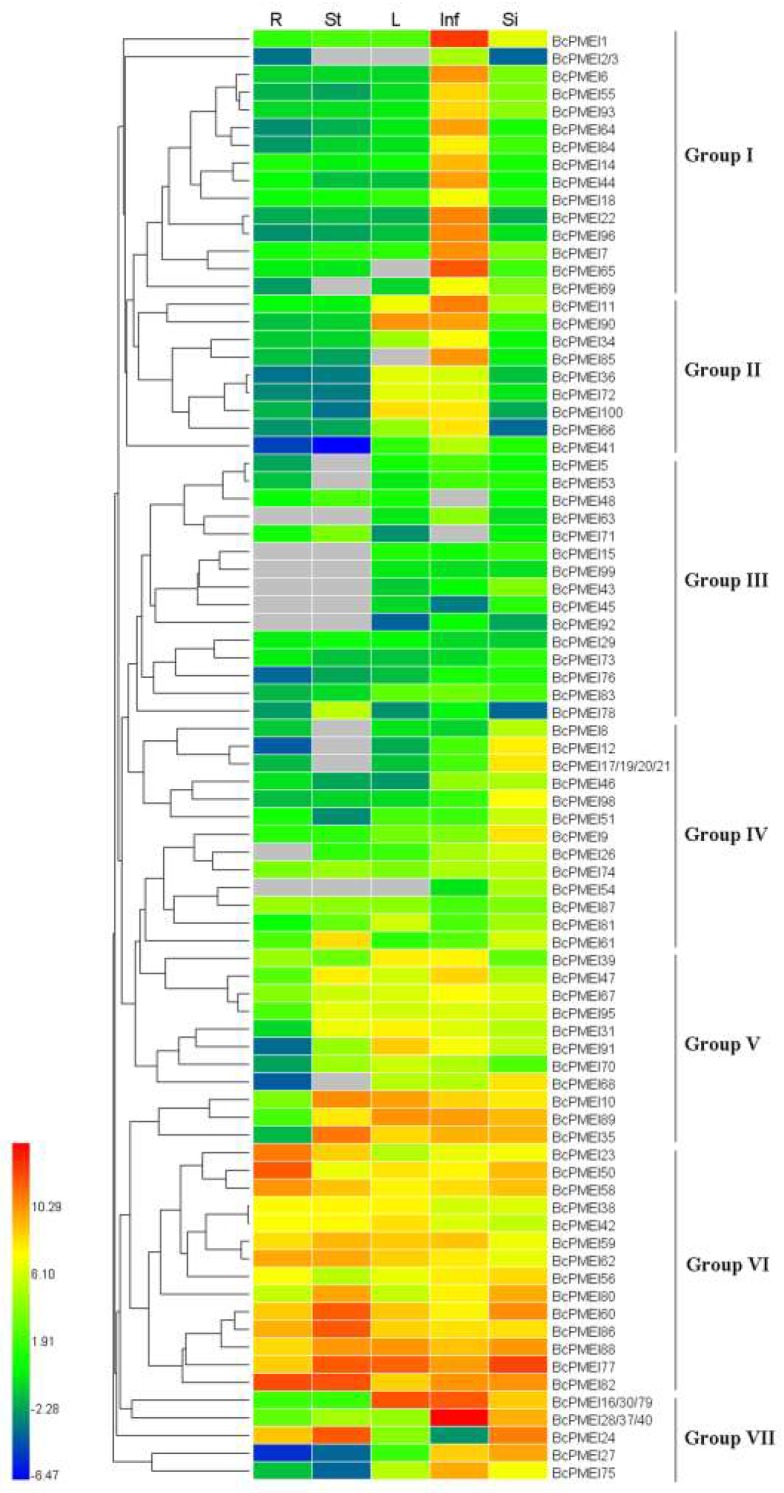
Hierarchical clustering and heat map representation displaying the expression profiles of *BcPMEIs* in root (R), stem (St), leaf (L), inflorescence (Inf), and silique (Si). The gene expression levels in the five tissues were explored by qRT-PCR. The scale bars on the left indicate relative expression level. The vertical dark bars on the right depict the seven groups of *BcPMEIs*. The grey boxes represent the undetectable expression.

To study the tissue-specific expression profiles of the 100 *BcPMEIs*, we conducted qRT-PCR in five major tissues, including roots, stems, leaves, inflorescences, and siliques. Because no expression of 10 *BcPMEIs* was detected in any of the five tissues, the expression patterns of 90 *BcPMEIs* were analyzed. The 90 *BcPMEIs* were classified into seven groups on the basis of their differential expression patterns (Figure 6). Groups I contained 16 *BcPMEIs* that were mainly expressed in inflorescences and group II contained nine *BcPMEIs* that were largely expressed in inflorescences and leaves. Group III included 15 *BcPMEIs* that showed quite low expression levels in all five tissues. The 16 *BcPMEIs* in group IV were largely expressed in siliques and 11 *BcPMEIs* in groups V were largely expressed in all five tissues except roots. Group VI contained 17 genes that had relatively higher expression in all five tissues. The other nine *BcPMEIs* belonged to group VII and were largely expressed in both inflorescences and siliques, although *BcPMEI16*/*30*/*79* was also highly expressed in leaves and *BcPMEI24* was weakly expressed in inflorescences and largely expressed in roots and stems.

**Figure 7:**
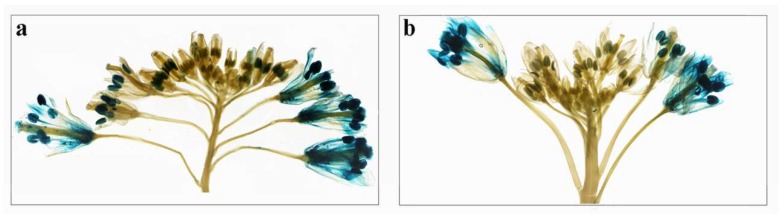
GUS staining of the *P_PMEI22_*::*GUS* inflorescence (**a**) and *P_PME44_*::*GUS* inflorescence (**b**).

The expression patterns of *BcPMEIs* in five tissues revealed that more than half of genes had a large or specific expression in reproductive tissues. To further study the roles that *BcPMEIs* might play in reproductive development, we selected two *BcPMEIs* that were largely and specifically expressed in inflorescences for the analysis of promoter::*GUS* fusion strategy in transgenic *Arabidopsis*. The results of histochemical staining in transgenic *Arabidopsis* plants displayed that the GUS activities were mainly detected in the late flower stages in the two *P_PMEI_*::*GUS* inflorescences (Figure 7). Particularly, very strong GUS activities were discovered in their mature anther and mature pollen grains, suggesting that they were likely to play roles in the later periods of pollen development. Moreover, the GUS staining could be seen in the sepals, petals, filaments, and pistils at the flower stages 12–14.

### 2.7. Roles of BcPMEIs in Pollen Development

**Figure 8:**
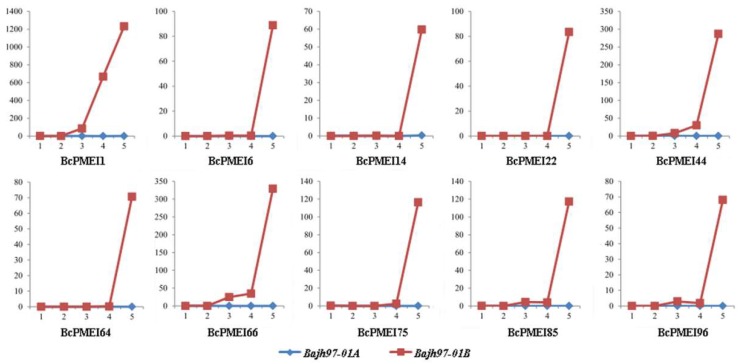
Expression profile analysis of representative *BcPMEIs* by the Illumina RNA-Seq data in A1–A5 and B1–B5. A1–A5 and B1–B5 present the five developmental stages of pollen in “*Bcajh97*-*01A*” and “*Bcajh97*-*01B*”, respectively.

**Figure 9:**
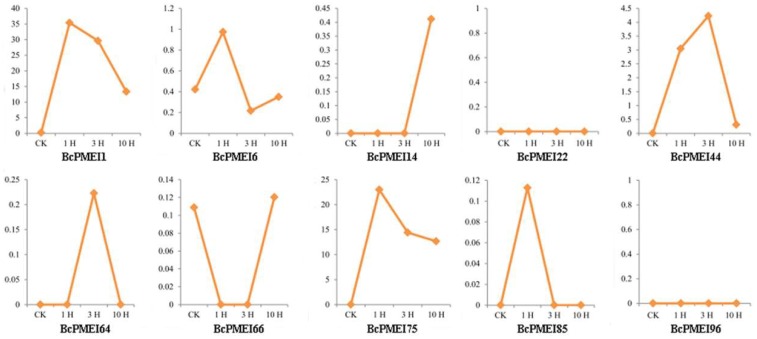
Expression profile analysis of representative *BcPMEIs* by the Illumina RNA-Seq data in unpollinated pistils (CK) and pollinated pistils at 1, 3, and 10 h after pollination (HAP).

The results of qRT-PCR showed that many *BcPMEIs* were specifically and highly expressed in inflorescences. Further, to explore the potential functions of *BcPMEIs* in pollen development, we used the Illumina RNA-Seq transcriptomic data of the “*Bcajh97*-*01A*/*B*” genic male sterile (GMS) line. In all, 10 *BcPMEIs* detected from the dataset showed high expressions in fertile flower buds but low or even no expression in sterile flower buds (Figure 7). All 10 *BcPMEIs* have the largest expression levels in stage V, and *BcPMEI1*, *BcPMEI44*, and *BcPMEI66* also have relatively low expression levels in stage III and stage IV (Figure 8). In general, the genes that are expressed in mature pollen can go on expressing in pollen germination and pollen tube growth. So, we analyzed the expression levels of the 10 *BcPMEIs* in the pistils of the sterile line that were collected at 1, 3, and 10 h after pollination (HAP) in a fertile line. Only *BcPMEI1* and *BcPMEI75* were detected to be largely expressed in the pollinated pistils in the whole fertilization processes while the remaining eight *BcPMEIs* had very low or no expression in the pollinated pistils (Figure 9), indicating that *BcPMEI1* and *BcPMEI75* might be involved in pollen germination and pollen tube growth.

### 2.8. Cis-Elements in the Putative Promoter Regions of BcPMEIs

To further study the transcriptional regulation and potential functions of *BcPMEIs*, we identified and analyzed the *cis*-regulatory elements in their promoter regions by the PlantCARE website. A total of 22 *cis*-regulatory elements involved in hormone treatments (ABA, ethylene, GA, MeJA, and salicylic acid) and stress tolerance (fungus, drought, high or low temperature, hypoxia, light, salt, and wound) were analyzed (Table S6). Both ABA (ABRE and CE3) and IAA (AuxRE, AuxRR-core, and TGA-element) related *cis*-elements were found in the promoter regions of 38 *BcPMEIs*. GA (GARE-motif, P-box, and TATC-box) and MeJA (TGACG-motif and CGTCA-motif) related *cis*-elements were discovered in 58 and 44 promoters of *BcPMEIs*, respectively. In addition, we also identified the ethylene-responsive elements (ERE) and salicylic acid responsive elements (TCA-element) in 32 and 61 promoters of *BcPMEIs*, respectively. In response to various biotic and abiotic stresses, 63 promoters containing *cis*-acting elements involved in heat stress responsiveness (HSE) and 35 promoters containing the low-temperature responsive (LTR) elements were identified. We also found the MYB binding site (MBS) involved in drought responsiveness in 58 promoters and the MRE related to light responsiveness in 19 promoters. The *cis*-element essential for anaerobic induction was detected in up to 71 promoters of *BcPMEIs*, whereas only 15 promoters covered the wound-responsive element. These results implied that *BcPMEIs* might play significant roles in hormone regulation and stress responses.

### 2.9. The Microarray Data Analysis of PMEIs in Arabidopsis

The *cis*-elements analysis reveals that *BcPMEIs* are essential in mediating the responses to hormones and stresses. The expression profiles of *AtPMEIs* under several different hormones (ABA, GA, IAA, and MeJA) (Table S7) and stresses (cold, drought, heat, oxidation, salt, and wounding) (Table S8) were downloaded from the Bio-Analytic Resource database [37]. Given the close relationships between *B. campestris* and *Arabidopsis*, these data were used to further explore the impacts of hormones and stresses on the expression levels of *PMEIs*. In all, 48 *AtPMEIs* were detected by expression profile tags. Following the exogenous hormone treatments, the expression levels of *AtPMEIs* were changed and their variation trends were varied (Figure S4). Some *PMEIs* were markedly changed at the three treatment time points. Following ABA treatment, the expression of *At1G14890*, *At4G12390*, *At4G25260*, and *At5G62360* were obviously downregulated while the expression of *At1G47960* was markedly upregulated. After GA treatment, the expression levels of *At5G62340* and *At5G62350* were decreased at 0.5 hours after treatment (HAT) and were remarkably increased at 1 and 3 HAT. The obvious upregulation of the expression of *At1G62770* and *At5G62340*, as well as the downregulation of the expression of *At1G23205*, *At4G12390*, and *At5G62360*, were observed in IAA treatment. Following MeJA treatment, the expression levels of *At1G70720*, *At5G62350*, and *At5G62360* were markedly increased and that of *At1G14890*, *At1G23205*, and *At4G12390* were decreased. Similarly, the microarray data analysis results of *PMEIs* under different stresses also revealed that many *PMEIs* are involved in the cold, drought, heat, oxidation, salt, and wounding regulation in *A. thaliana*.

## 3. Discussion

PMEIs play crucial roles in pectin remodeling and disassembly by regulating the activity of PMEs in plant growth and development [38]. In 1990, PMEIs were first found in kiwi (*Actinidia deliciosa*) [39]. To date, *PMEIs* have been investigated in many plant species, such as *Arabidopsis*, broccoli, grape, maize, pepper, and tomato, and were been identified to be a large multigene family [9,11,18,32,40,41,42]. In this study, 100 PMEIs were identified in *B. campestris* (Table S1). The number of *PMEIs* changed greatly in different plants that more *PMEIs* were detected in dicots than that in monocots with 95 members in flax, 78 in *Arabidopsis*, and 49 in rice [29,30,31]. This phenomenon is in accordance with the finding that pectin is more abundant in dicots than in monocots [43,44]. 

As has been reported, *A. thaliana*, the model plant, has experienced three WGD events: a γ event shared with most dicots and two subsequent genome duplications (α and β) shared with other members of the order Brassicales [25]. *B. campestris*, which shares a common ancestor with *Arabidopsis*, also experienced the three abovementioned WGD events. Approximately 13 to 17 MYA, *B. campestris* underwent another WGT event, resulting in the divergence of the genome between it and *A. thaliana* [27,28]. In this study, the number of PMEIs identified in *B. campestris* (100) was higher than that in *A. thaliana* (78). The *PMEIs* in *B. campestris* were distributed in the 10 chromosomes unevenly and up to 80 syntenic PMEI ortholog pairs were identified between the genome of *B. campestris* and *A. thaliana* (Figure 3). These findings provide the evidence that WGT results in the expansion of the PMEI gene family in *B. campestris*. TD is another crucial mode of gene expansion. Up to 2137, 1569, 1751, and 1135 tandemly duplicated clusters were discovered in *B. campestris*, *A. thaliana*, *A. lyrata*, and *T. parvula*, respectively [45]. Among the 100 *BcPMEIs*, we detected 9 tandem arrays containing 20 *BcPMEIs* (Figure 2), indicating that TD also contributes a lot to the expansion of PMEI gene family in *B. campestris*. WGD and TD are the main ways to account for the expansion of *BcPMEIs*, similar to the PMEI family in *A. thaliana* [32].

During the evolution of plants, diploidization always occurs after WGD and is usually accompanied by substantial gene fractionation [46]. In *Arabidopsis*, 27,411 genes were identified, so the *B. campestris* genome was supposed to have approximately 90,000 genes because of the WGT event. However, only 41,174 members were detected in the newly formed hexaploid in fact [26]. This case is representative of the considerable gene fractionation that happens following polyploid formation in eukaryotes [47,48,49]. In our work, only 100 *PMEIs* were identified in the genome of *B. campestris*, much less than the three times of 78 *PMEIs* in *A. thaliana* (Table S1), suggesting that many *BcPMEIs* were lost after WGT. The collapse of the *BcPMEI* gene complement might result from the genome-level gene loss. To investigate the fractionation extent of *BcPMEIs* after WGT, we calculated the reserved rate of *BcPMEIs* and compared it with the reserved rates of two other gene sets: a set of 458 randomly selected genes and 458 core eukaryotic genes (Figure 4). The result manifested that *BcPMEIs* were preferentially retained with a retention proportion of 52% (Figure 4a). This phenomenon is consistent with the gene dosage hypothesis, which proposes that genes whose products interact either with other proteins or in networks are more likely to be retained, to avoid the stoichiometric imbalances among the products [50,51]. In the metabolic network of pectin in cell walls, PMEIs play a crucial part and are highly connected with other enzymes, including PMEs, polygalacturonases (PGs), and pectate lyases (PLs) [52]. In *B. campestris*, the three subgenomes, namely LF, MF1, and MF2, were named according to the extent of gene fractionation relative to *A. thaliana* since 13–17 MYA. Approximately 70%, 46%, and 36% of the genes found in *Arabidopsis* were retained in LF, MF1, and MF2 subgenomes, respectively [26]. In this work, *BcPMEIs* were also identified to be biasedly retained (Figure 4c). More *BcPMEIs* were reserved in LF subgenome than that in MF subgenomes, and more *BcPMEIs* were reserved in MF1 subgenome than that in MF2 subgenome, in accordance with the previous study [26]. These results can be explained by a ‘two-step theory’, which supposes that MF1 and MF2 subgenomes underwent two rounds of gene fractionation and LF subgenome only under one round, therefore MF1 and MF2 subgenomes lost more genes than LF subgenome [53]. 

To explore the selection type of genes after duplication, the *Ka* and *Ks* modes of ortholog and paralog pairs were analyzed. Taking advantage of the commonly used estimate of the mutational rate of 1.5 synonymous substitutions per 10^8^ years, we calculated the divergence time [54]. The average duplication time of the 84 pairs of orthologs was 16.67 MYA (Figure 5a, Table S4), which was in accordance with the divergence time of *B. campestris* and *Arabidopsis* [26]. Additionally, among the three subgenomes, the average duplication time of the 52 BcPMEI paralog pairs was 16.17 MYA (Figure 5c, Table S5), in accordance with the formation time of the three subgenomes in *B. campestris*. Most of the homologous gene pairs experienced purifying selection, indicating that these genes were strongly controlled in evolution. Only one ortholog pair and one paralog pair have a ω ratio larger than 1 (Figure 5b,d), suggesting that novel functions were likely to generate among these genes. This is the same as some other gene families in plants, such as TCS of tomato, GRAS of *Medicago truncatula*, and NF-YB of *Gossypium hirsutum*, of which most homolog pairs evolve through purifying selection and few or even no gene experience positive selection [55,56,57].

Many studies have demonstrated that *PMEIs* play important parts in plant growth and development. *AtPMEI1* and *AtPMEI2*, mainly detected in anthers and pollen, have an important function during pollen development [41]. *AtPMEI4* can regulate the growth acceleration of the dark-grown seedlings [10] and *AtPMEI6* is involved in seed maturation and germination [16]. Generally, gene expression patterns can offer helpful information for studying gene functions. In our work, the expression profiles of the 100 *BcPMEIs* in five different tissues were analyzed and 90% of the *BcPMEIs* could be detected in at least one tissue (Figure 6). As many *BcPMEIs* displayed a high and/or specific expression level in inflorescences, we conducted a further analysis to search candidate *BcPMEIs* that may be related to pollen development using the RNA-seq data (A1–A5, B1–B5). As shown in Figure 8, 10 *BcPMEIs* were identified to have specific expressions in the fifth stage of the fertile flower buds except for three genes, which were also expressed in the third and fourth stages (Figure 8). This finding is in accordance with the results of GUS staining (Figure 7). Also, among the 10 *BcPMEIs*, *BcPMEI44*, *BcPMEI55*, and *BcPMEI73* might be closely related to male sterility [14]. Considering that genes highly expressed in mature pollen generally keep being expressed in pollen germination and pollen tube growth, we also explored the expression levels of the 10 genes in pistils at 1, 3, and 10 HAP. Two members, *BcPMEI1* and *BcPMEI75*, were identified to be expressed in pistils in the whole process of fertilization (Figure 9), suggesting that they might be involved in the pollen germination and pollen tube growth. BoPMEI1, specifically expressed in mature pollen and pollen tubes, can suppress the expression of its orthologous gene *At1G10770* and causes the male sterility of the transgenic *Arabidopsis* by impairing pollen tube growth [9]. Coincidentally, *At1G10770* is also the orthologous gene of *BcPMEI1*. We speculate that *BcPMEI1* is more likely to play critical parts in the pollen tube growth of *B. campestris*. Pollen tube growth contains cell wall synthesis and expansion, which are influenced by the PME activity [58]. The activity of PMEs is regulated by PMEIs, hence PMEIs can function in pollen tube growth by affecting the cell wall stability [59].

Increasing evidence proved that *PMEIs* are involved in hormone regulation and stress tolerance by controlling the biophysical properties of plant cell walls. Transgenic *Arabidopsis* plants with ectopic *CaPMEI1* expression show reduced sensitivity to oxidative and drought stresses [18]. An *Arabidopsis* mutant with T-DNA insertion in the promoter region of a PMEI gene (*AtPMEI10*) presents enhanced resistance to salinity stress [60]. When overexpressed an *Arabidopsis PMEI* gene, *AtPMEI5*, the PME activity of transgenic seed is decreased and the degree of cell wall pectin methylesterification is increased; the speed of seed germination is faster and the sensitivity of seed to the inhibitory effects of ABA on germination is lowered [10]. AtPMEI10, AtPMEI11, and AtPMEI12 were identified as functional PME inhibitors that can control the integrity of cell walls to fight against the *Botrytis* attack; the expressions of AtPMEI11 and AtPMEI12 are strictly mediated by Jasmonic Acid and Ethylene signaling [13]. In this work, many *cis*-regulatory elements related to the responses of different hormone treatments including ABA, IAA, MeJA, and GA, and different stresses, including anaerobism, cold, heat, drought, salt, and fungus were detected (Table S6). *BcPMEI17* has up to 27 related *cis*-elements, with 10 *cis*-elements involved in MeJA responsiveness and 4 *cis*-elements involved in heat responsiveness. Among the 26 *cis*-elements detected in the promoter of *BcPMEI90*, 11 *cis*-elements were related to anaerobic responsiveness and 4 *cis*-elements were related to ABA responsiveness. In addition, the analysis results of the microarray data of 48 *AtPMEIs* revealed that many PMEIs can be strongly induced in response to some exogenous hormones and stresses (Tables S8 and S9). However, further researches are required to investigate the detailed regulatory mechanisms of *PMEIs* in *B. campestris* under different stimuli.

## 4. Materials and Methods

### 4.1. Plant Materials

The Chinese cabbage-pak-choi ‘Aijiaohuang’ (*B. campestris* L. ssp. *chinensis* Makino cv. Aijiaohuang) named ‘*Bajh97*-*01A*/*B*’ provides the materials used for the expression analysis in this study. It was a GMS system and was developed by continuous backcross for more than 10 years. The progenies of ‘*Bajh97*-*01A*/*B*’ were segregated into a homozygous male sterile line (*Bcajh97*-*01A*) and a heterozygous male fertile line (*Bcajh97*-*01B*) with a 1:1 ratio. The only difference presented between the sibling (sister) lines is that ‘*Bcajh97*-*01A*’ is in a complete absence of mature pollen compared with that of ‘*Bcajh97*-*01B*’ [61,62]. This feature makes ‘*Bajh97*-*01A*/*B*’ become an ideal material to explore the genes closely related to pollen development. 

The ‘*Bcajh97*-*01A*/*B*’ was grown under natural conditions in an experimental farm at Zhejiang University, China. To study the expression profiles of *BcPMEIs* in roots, stems, leaves, inflorescences, and siliques of ‘*Bcajh97*-*01B*’, the five organs were sampled from 15 individual plants at the flowering stage. Before RNA extraction, all the materials were frozen in liquid nitrogen immediately after collected and stored at –80 °C.

According to the results of the cytological examination described in previous studies [63,64], the flower buds were classified into five stages: stage I, pollen mother cell stage; stage II, tetrad stage; stage III, uninucleate microspore stage; stage IV, binucleate microspore stage; and stage V, mature pollen stage. In ‘*Bcajh97*-*01A*’ and ‘*Bcajh97*-*01B*’ plants, the flower buds at five pollen development stages were named as A1–A5 and B1–B5, respectively. In addition, pistils in the sterile ‘*Bcajh97*-*01A*’ pollinated by the pollen of fertile plants were sampled at 1, 3, and 10 HAP [64]. To study the potential functions of *BcPMEIs* in the pollen development of *B. campestris*, we used the Illumina RNA-seq data of ‘*Bcajh97*-*01A*/*B*’. Of the dataset, A1–A5, B1–B5, the unpollinated pistils, and the pollinated pistils sampled at 1, 3, and 10 HAP were analyzed.

### 4.2. Identification of PMEIs in B. campestris

The genome-wide protein sequences of *B. campestris* were retrieved from the *Brassica* database (BRAD, http://brassicadb.org/brad/, V1.5) [65]. The hidden Markov model (HMM) file of PMEI domain (PF04043) was obtained from Pfam 31.0 (http://pfam.xfam.org/) [66] and was used as a query in the following HMM analysis. Then, the HMMER V3.0 software was used to search candidate PMEI protein sequences in the *B. campestris* protein genome with default values. Meanwhile, the protein sequences of 78 *Arabidopsis thaliana PMEIs* (*AtPMEIs*) [31] and 49 *Oryza sativa PMEIs* (*OsPMEIs*) were downloaded from The Arabidopsis Information Resource (TAIR, http://www.arabidopsis.org/) and Rice Genome Annotation Project (http://rice.plantbiology.msu.edu/) [67], respectively, and were used as queries to conduct the BLASTP search in BRAD with default parameters. Finally, we used the SMART (http://smart.embl-heidelberg.de/) [68] and Pfam databases to further confirm the presence of PMEI domain (PF04043). The identified *BcPMEIs* were named based on their orders in chromosomes or scaffolds, from top to bottom. 

### 4.3. Phylogenetic, Gene Structural, and Physicochemical Properties Analysis

The protein, coding, and genomic sequences of *BcPMEIs* used in the following analysis were retrieved from BRAD [65]. The full-length protein sequence alignments of PMEIs were conducted by the ClustalW program of MEGA6.0 with the default parameters [69,70]. Then the MEGA6.0 software was used to construct the phylogenetic tree with the statistical method of neighbor-joining (NJ), the bootstrap replications of 1000, the model/method of passion, the rates among sites of uniform, and the gaps/missing data treatment of pairwise deletion. The exon–intron structures of *BcPMEIs* were analyzed by Gene Structure Display Server 2.0 (GSDS 2.0) (http://gsds.cbi.pku.edu.cn/index.php) [71]. The intron phases were also analyzed with phases 0, 1, and 2, which were assigned to the introns located between codons, the introns located between the first and second nucleotides of a codon, and the introns located between the second and third nucleotides of a codon, respectively. Plant-mPLoc (http://www.csbio.sjtu.edu.cn/bioinf/plant-multi/) was used to analyze the subcellular localization of PMEI proteins [72]. PROTEIN CALCULATOR v3.4 (http://protcalc.sourceforge.net/) was used to compute theoretical isoelectric point and molecular weight and SMART was used to analyze signal peptide sequence. TMHMM Server V2.0 (http://www.cbs.dtu.dk/services/TMHMM/) [73] was taken advantage to predict transmembrane helices (TMHs) in BcPMEI proteins. 

### 4.4. Motif Recognition, Putative Promoter Region Analysis, and Promoter::GUS Fusion Construction

The online program of Multiple Em for Motif Elicitation (MEME Version 4.11.4) (http://meme-suite.org/tools/meme) [74] was used to explore the distributions of BcPMEI protein motifs and the parameters were set up as below: number of repetitions, any; width of the motif, 6–50; and maximum number of motifs, 10. To analyze the *cis*-elements in promoter sequences of *BcPMEIs*, the upstream sequences (1.5 kb) of the initiation codon (ATG) for *BcPMEIs* were extracted from BRAD. Then the PlantCARE website (http://bioinformatics.psb.ugent.be/webtools/plantcare/html/) [75] was used to identify the *cis*-elements in promoter regions. The predicted promoter sequences of two *BcPMEIs* were amplified from the genomic DNAs of *B. campestris* using the gene-specific primers (Table S9) and cloned into the binary vector *pBI101*. The resulting constructs were transferred into *Agrobacterium tumefaciens* and then transformed into *Arabidopsis*. The inflorescences of the transgenic *Arabidopsis* plants were used to detect GUS activity as previously described [76]. Stages of flower development in *Arabidopsis* were confirmed on the basis of a previous study [77]. 

### 4.5. Chromosome Location, Synteny, Retained Rate, and Evolutionary Analysis

The precise positions of *PMEIs* on the *B. campestris* chromosomes were derived from the *Brassica* Genome Browse (http://brassicadb.org/cgi-bin/gbrowse/Brassica_v1.5/) and the Mapinspect and Adobe Photoshop CC software were used to draw the map. The chromosomal positions of *AtPMEIs* were obtained from TAIR. The syntenic relationships between *AtPMEIs* and *BcPMEIs* were identified by searching “syntenic gene” (http://brassicadb.org/brad/searchSyntenytPCK.php) in BRAD [33]. Furthermore, the information of 24 conserved collinear blocks of ancestral karyotype (A-K) in *B. campestris* and *Arabidopsis* was acquired from a previous research [78]. Circos 5.05 software was used to display the colinearity of *PMEIs* in or between *B. campestris* and *A. thaliana* genomes [79]. To compute the retention rates, we used the term “locus” to replace “gene”, which can exclude the interferences of tandem duplication after WGT [80]. The full-length amino acid sequence alignments were performed by Clustal Omega (http://www.ebi.ac.uk/Tools/msa/clustalo/) and then *Ka, Ks* and ω ratio (*Ka*/*Ks*) were counted using the PAL2NAL by the codeml program in PAML (http://www.bork.embl.de/pal2nal/index.cgi?example=Yes#RunP2N) [81,82]. The divergence time was calculated by the following equation: T = *Ks*/2R (R = 1.5 × 10^−8^ for dicotyledonous plants) [54]. 

### 4.6. RNA Isolation and Expression Profile Analysis

The total RNA was extracted from roots, stems, leaves, inflorescences, and siliques using Trizol reagent (Invitrogen, Carlsbad, CA, USA) treated with DNAase on the basis of the manufacturer-recommended protocol. The PrimerScript RT reagent kit (TaKaRa, Shiga, Japan) was used to reverse-transcribe total RNA into the first strand of complementary DNA. The gene expression was measured by applying diluted cDNA in a SYBR Premix Ex Taq Kit (TOYOBO, Osaka, Japan) with CFX96 Real-Time System (Bio-Rad, Hercules, CA, USA). *BcUBC10* was used as internal control [83]. The gene-specific primers listed in Table S10 were designed by the Primer Premier 5.0 software and the BLASTN page of BRAD was used to verify the specificity of each primer. QRT-PCR was carried out in triplicate. The qRT-PCR conditions were optimized to consist of an initial denaturation for 30 s at 95 °C, followed by 40 cycles of 5 s at 95 °C and 30 s at 55 °C, and at the end, 1 cycle of 10 s at 95 °C, 5 s at 65 °C and 5 s at 95 °C. The 2^−ΔΔCt^ method was applied to compute the relative expression levels of different genes [84] and the Heatmap Illustrator (HemI 1.0) (http://hemi.biocuckoo.org/index.php) was used to make the results of qRT-PCR in a heat map [85]. In addition, to know the impacts of different hormones and stresses on the expression levels of *PMEIs*, the values of *AtPMEIs* treated with 10 µM ABA, 1 µM GA, 1 µM IAA, 10 µM MeJA, cold, salt, heat, drought, oxidation, and wounding were download from the Bio-Analytic Resource database (http://bar.utoronto.ca/affydb/cgi-bin/affy_db_exprss_browser_in.cgi) [37].

## 5. Conclusions

In this study, a genome-wide analysis of the PMEI gene family in *B. campestris* was carried out on the base of publicly available genome data. A total of 100 *BcPMEIs* were identified with 96 members unevenly distributed on 10 chromosomes and four members distributed on different scaffolds. In the evolutionary process, the PMEI gene family of *B. campestris* was expanded mainly because of WGT and TD. During diploidization after WGT, *BcPMEIs* were preferentially and biasedly retained. The evolution analysis suggested that most of the duplicated genes evolved through purifying selection, indicating the strong controls among these genes. In the analyses of qRT-PCR and RNA-seq data, 10 *BcPMEIs* were identified to be involved in pollen development, among which two genes might play important roles in pollen germination and pollen tube growth. Furthermore, the promoter analysis results suggested that *BcPMEIs* might be closely related with the responses to multiple stimuli. This work will be beneficial to understand the molecular evolution of *BcPMEIs* and select proper candidate genes for further functional characterization.

## Supplementary Materials

Supplementary materials can be found at http://www.mdpi.com/1422-0067/19/5/1338/s1. Figure S1: Phylogenetic relationships of the 100 BcPMEIs. The phylogenetic tree was carried out using MEGA 6 by the neighbor-joining (NJ) analysis with 1000 replicates. Bootstrap values less than 50 are not shown. This tree was classified into five phylogenetic subgroups marked with different colors. Figure S2: The sequences of 10 motifs identified from the 100 BcPMEIs. Figure S3: Box plots of *Ks* values (a) and *Ka*/*Ks* values (b) of the PMEI ortholog pairs between each of the three *Brassica campestris* subgenomes and *Arabidopsis*. Figure S4: Relative expression levels of *AtPMEIs* under ABA (a), GA (b), IAA (c), and MeJA (d) treatments, respectively. Table S1: Basic information of the PMEI gene family in *Brassica campestris*. Table S2: The homologous relationships between *BcPMEIs* and *AtPMEIs*. *BcPMEIs* in black have syntenic relationships to their corresponding *AtPMEIs*. *BcPMEIs* in red are nonsyntenic orthologs to their corresponding *AtPMEIs*. *BcPMEIs* in bold are not sure which subgenomes they are on. tPCK Chr, Chromosome of translocation Proto-Calepineae Karyotype, ancestral genome of *Brassica* species. Table S3: Orthologous genes of *AtPMEIs* and *BcPMEIs* in sequenced Brassicaceae species. *Aar*, *Aethionema arabicum*; *Aly*, *Arabidopsis lyrata*; *Ath*, *Arabidopsis thaliana*; *Bna*, *Brassica napus*; *Bol*, *Brassica oleracea*; *Bra*, *Brassica rapa*; *Cru*, *Capsella rubella*; *Csa*, *Camelina sativa*; *Lal*, *Leavenworthia alabamica*; *Sir*, *Sisymbrium irio*; *Spa*, *Schrenkiella parvula*; *Tha*, *Thellungiella halophila*; *Tsa*, *Thellungiella salsuginea*. Table S4: *Ka*, *Ks*, *Ka*/*Ks*, and divergent time calculation of the PMEI ortholog pairs between the *Brassica campestris* and *Arabidopsis thaliana* genomes. Table S5: *Ka*, *Ks*, *Ka*/*Ks*, and divergent time calculation of PMEI paralog pairs in *Brassica campestris* genome. Table S6: *Cis*-element analysis in the promoter regions of *BcPMEIs*. Table S7: Relative expression levels of *AtPMEIs* under different hormone treatments. Table S8: Relative expression levels of *AtPMEIs* under different stresses. Table S9: Gene primers used in the promoter amplification of *BcPMEIs*. Table S10: Gene primers for the qRT-PCR analysis of *PMEIs* in *Brassica campestris*.

## Abbreviations

AtPMEIs	*Arabidopsis thaliana* PMEI genes
BcPMEIs	*Brassica campestris* PMEI genes
BRAD	brassica database
GMS	genic male sterile
HAP	hour after pollination
HAT	hour after treatment
HMM	hidden Markov model
HG	homogalacturonan
*Ka*	nonsynonymous substitution rate
*Ks*	synonymous substitution rate
LF	least fractionated subgenome
MEME	multiple em for motif elicitation
MF1	medium fractionated subgenome
MF2	most fractionated subgenome
MYA	million years ago
NJ	neighbor-joining
PGs	polygalacturonases
PLs	pectate lyases
PMEs	pectin methylesterases
PMEIs	pectin methylesterase inhibitors
qRT-PCR	quantitative real-time PCR
RGI	rhamnogalacturonan I
RGII	rhamnogalacturonan II
TAIR	the arabidopsis information resource
TD	tandem duplication
WGT	whole genome triplication
WGD	whole genome duplication
XGA	xylogalacturonan
