# Supplementary figures and images for: Genome-Wide Identification, Molecular Evolution, and Expression Profiling Analysis of Pectin Methylesterase Inhibitor Genes in Brassica campestris ssp. chinensis
Source: Int J Mol Sci. 2018 May 2;19(5):1338. doi: 10.3390/ijms19051338 (PMC5983585; doi:10.3390/ijms19051338)

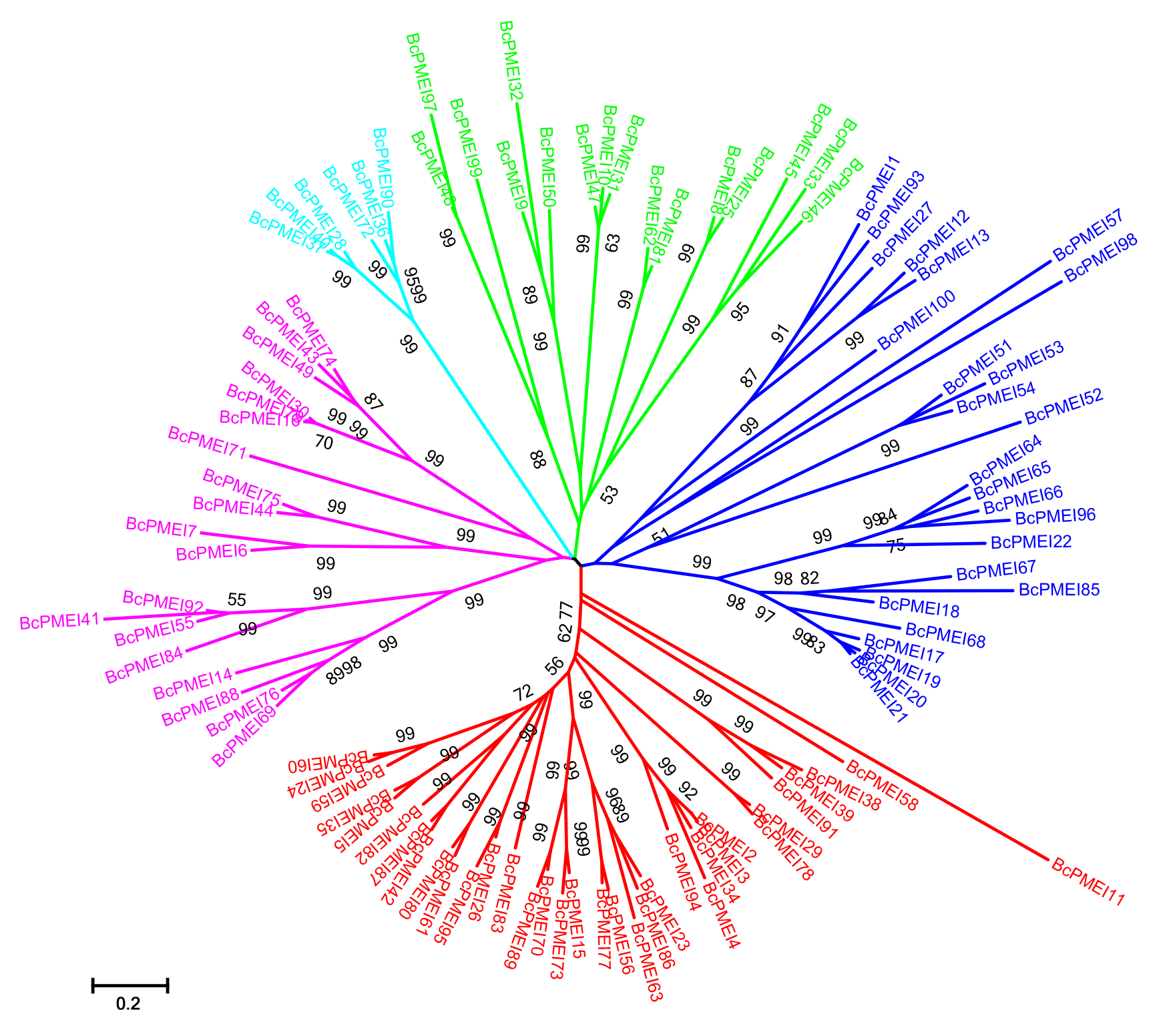

Supplement: Supplementary file 1 [file ijms-19-01338-s001.zip › Supplementary materials/Figure S1.tif]

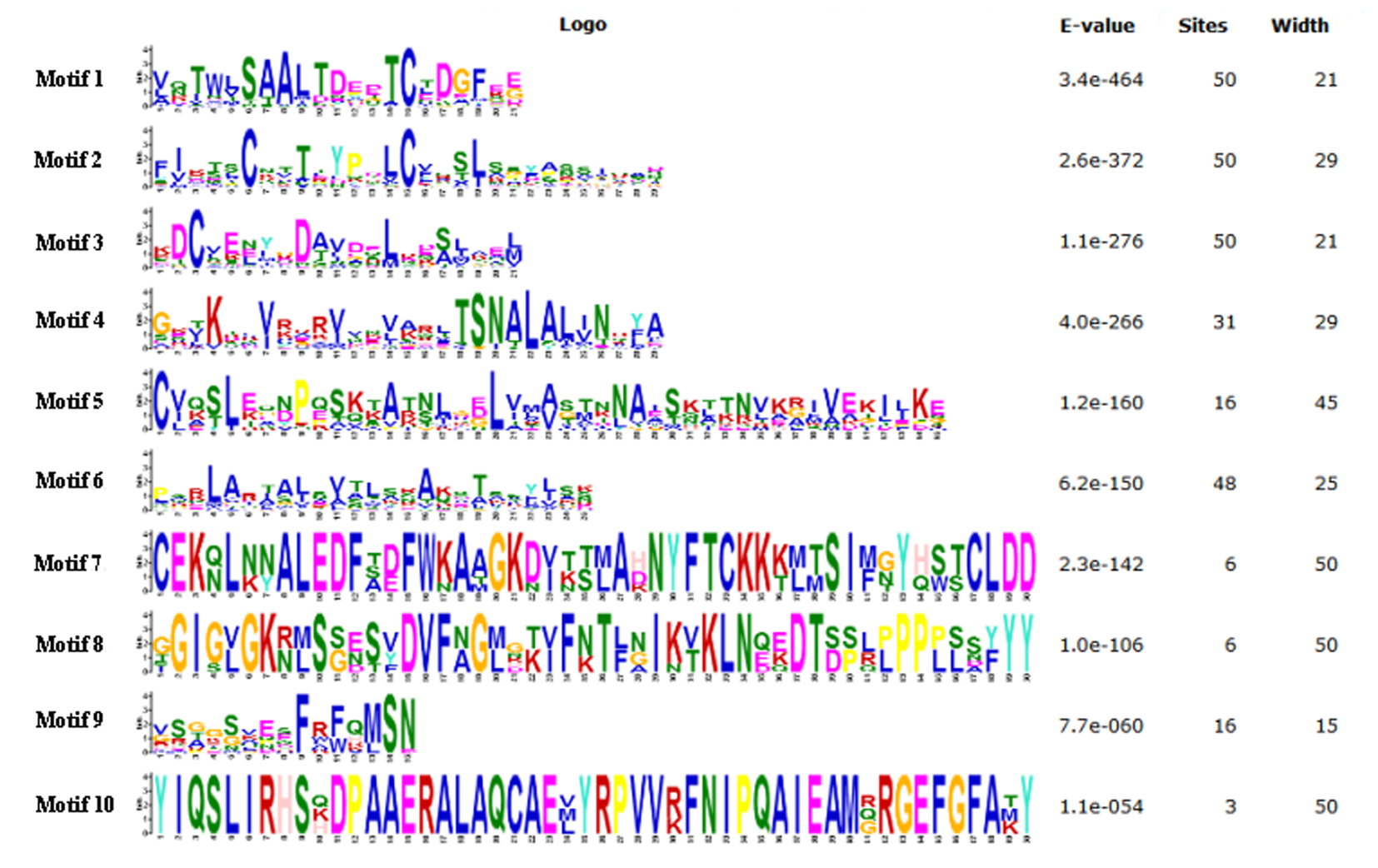

Supplement: Supplementary file 1 [file ijms-19-01338-s001.zip › Supplementary materials/Figure S2.tif]

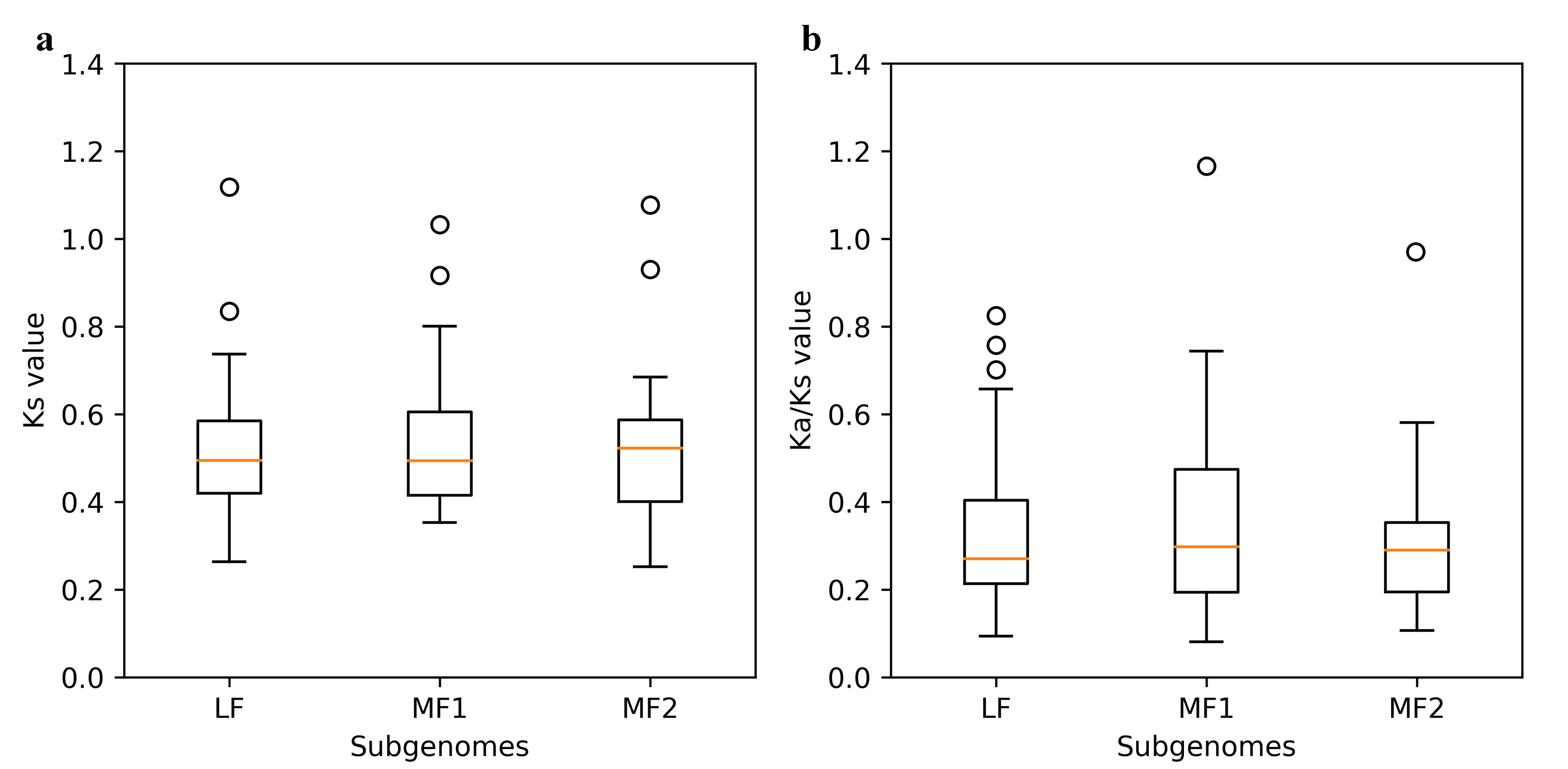

Supplement: Supplementary file 1 [file ijms-19-01338-s001.zip › Supplementary materials/Figure S3.tif]

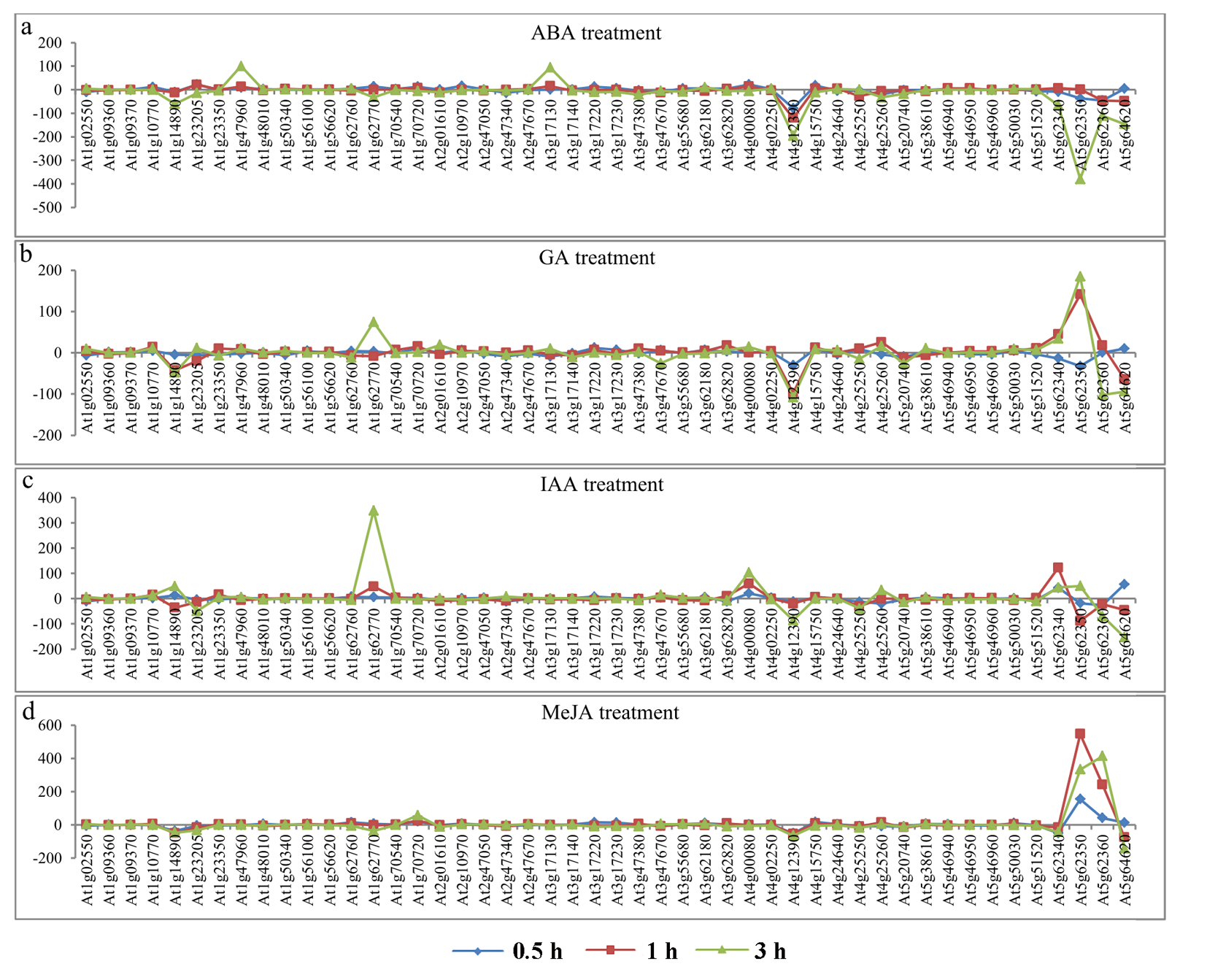

Supplement: Supplementary file 1 [file ijms-19-01338-s001.zip › Supplementary materials/Figure S4.tif]
